# Abundance of cell‐free mitochondrial DNA in spent culture medium associated with morphokinetics and blastocyst collapse of expanded blastocysts

**DOI:** 10.1002/rmb2.12344

**Published:** 2020-08-11

**Authors:** Mitsuru Kobayashi, Junichi Kobayashi, Koumei Shirasuna, Hisataka Iwata

**Affiliations:** ^1^ Department of Animal Science Tokyo University of Agriculture Atsugi Japan; ^2^ Kanagawa Ladies Clinic Yokohama Japan

**Keywords:** blastocyst collapse, cell‐free DNA, embryo selection, mitochondria, time‐lapse incubator

## Abstract

**Purpose:**

This retrospective observational study investigated relationships between the abundance of cell‐free mitochondrial DNA (cf‐mtDNA) in spent culture medium (SCM) of human‐expanded blastocysts and their morphokinetics to address the question of whether the abundance of cf‐mtDNA in SCM could predict the quality of blastocysts.

**Methods:**

Embryos (n = 53) were individually cultured in a time‐lapse incubator until they reached the expanded blastocyst stage (5 or 6 days), following which copy numbers of cf‐mtDNA in SCM (20 μL) of expanded blastocysts were determined using real‐time PCR.

**Results:**

The duration between start of blastulation to expanded blastocyst (tEB–tSB) and between that of the blastocyst stage to expanded blastocyst (tEB–tB) significantly and positively correlated with the abundance of cf‐mtDNA in the SCM (tEB–tSB: r = .46; *P* < .01; tEB–tB: r = .47; *P* < .01). The abundance of cf‐mtDNA in the SCM was significantly greater in blastocysts with blastocyst collapse (BC), than without BC, and significantly and positively correlated with the number of BC.

**Conclusions:**

The abundance of cf‐mtDNA in the SCM was associated with expansion duration and BC. Thus, cf‐mtDNA abundance in the SCM serves as a marker to predict the quality of expanded blastocysts.

## Graphical abstract

The abundance of cell free‐mitochondrial DNA in the spent culture medium is associated with expansion duration and presence of blastocyst collapse.

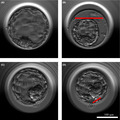

## 1 INTRODUCTION

Single blastocyst transfer (SBT) is a useful method of reducing the likelihood of multiple pregnancies that place patients at risk. Morphological evaluation with microscopy is a typical noninvasive approach used in embryo selection.^1^, ^2^ Although straightforward, the accuracy of the evaluated outcomes depends on observer skill.^3^ In addition to morphological evaluation, morphokinetic data of embryos, including profiles of embryo cleavage and developmental speed obtained using a time‐lapse incubator, closely relate to clinical pregnancy rates (CPR), euploidy, and live birth rates (LBR).^4^, ^5^, ^6^, ^7^ A pre‐implantation genetic test (PGT) has recently been developed to select chromosomally normal embryos before SBT, which improves CPR and reduces spontaneous abortion rates. Although these evaluation methods are useful for embryo selection, the LBR after elective SBT remains low, at 40%–70%.^8^, ^9^, ^10^ Therefore, other parameters are needed for embryo evaluation. Spent culture medium (SCM) contains cell‐free DNA (cf‐DNA) derived from embryos and might serve as a noninvasive marker of embryo status. However, a study of the relationship between cf‐DNA derived from embryonic nuclei and aneuploidy found that contamination with embryo‐associated structures led to a low probability of cf‐DNA serving as a marker of embryo status.^11^ Moreover, more mitochondrial (mtDNA) than nuclear DNA is detected in SCM because cells and embryos harbor more copies of the mitochondrial, than the nuclear genome. Sequencing of DNA extracted from porcine follicular fluid and real‐time PCR of DNA extracted from SCM of porcine granulosa cells has revealed that the abundance of cf‐mtDNA in SCM exceeds that of cf‐nucleic DNA by factors ranging from the tens to the hundreds,^12^, ^13^ indicating that cf‐mtDNA in SCM could serve as a marker for embryonic evaluation. The abundance of cf‐mtDNA in SCM reflects the mitochondrial status of porcine granulosa cells and bovine early cleaved embryos.^14^, ^15^ Furthermore, the abundance of cf‐mtDNA in SCM of human embryos (day 3 post‐insemination) reflects a higher developmental rate and raises pregnancy expectations.^16^ However, how abundance of cf‐mtDNA in SCM relates to the developmental events and quality of the blastocysts remains unknown. In the present study, we assessed embryo development till the expanded blastocyst stage using a time‐lapse incubator to determine cf‐mtDNA abundance in SCM, and its relationship to morphokinetic data.

## 2 MATERIALS AND METHODS

### 2.1 Ethical approval

The Institutional Review Board at Kanagawa Ladies Clinic approved this retrospective observational study (Approval no. 003; 2017). All involved patients provided informed consent to participate in this study, following which samples were collected and analyzed.

### 2.2 Participating patients and ovarian stimulation

Twenty couples underwent in vitro fertilization (IVF) at the Kanagawa Ladies Clinic in Kanagawa, Japan, during 2019. All patients were treated with an ovarian stimulation protocol using a gonadotropin releasing hormone (GnRH) agonist or antagonist. The effects of follicle‐stimulating hormone (FSH) stimulation were monitored by measuring serum estradiol (E2) levels and follicle growth. Cycles without proper follicle growth and E2 elevation were excluded from further treatment. Human chorionic gonadotropin (hCG) was administered when follicles reached a diameter of ≥18 mm, and oocytes were retrieved 35‐36 h later, rinsed with Sydney IVF Fertilization Medium (Cook Medical), and incubated until insemination.

### 2.3 Insemination and embryo culture, frozen‐thawed transfer

We counted the numbers, assessed the motility, and measured concentrations of spermatozoa in fresh semen, and selected those with high motility based on swim‐up protocols. Using the standard IVF protocol, oocytes were co‐incubated with 5 × 10^6^/mL spermatozoa in fertilization medium for 5 hours, and then cumulus cells were denuded from the oocytes by mechanical pipetting. The cumulus cells were denuded from the oocytes by mechanical pipetting of 40 IU/mL of hyaluronidase (ORIGIO, CooperSurgical) before intra‐cytoplasmic sperm injection (ICSI). After oocytes denudation, the absence of any cumulus cells attached to the zona pellucida (ZP) was confirmed by microscopy. ICSI proceeded in Sydney IVF Gamete Buffer containing HEPES (Cook Medical) using a PMM‐150HJ PIEZO micro manipulator (PRIMETECH Corp.). After insemination, zygotes were placed individually in the wells of equilibrated EmbryoSlide culture dishes (Vitrolife, Göteborg, Sweden) with 25 µL of global total medium (LifeGlobal, CooperSurgical) and covered by OVOIL™ (Vitrolife). The slides were incubated at 37°C under a 6% CO_2_, 5% O_2_, 89% N_2_ atmosphere in an EmbryoScope time‐lapse incubator (Vitrolife). Expanded blastocysts were vitrified on day 5 or 6 with a vitrification media kit and a Cryotop device (Kitazato). Thawing procedures were performed with a thawing media Kit (Kitazato), and laser‐assisted hatching was performed during the thawing procedure. Frozen‐thawed embryo transfers were conducted with endometrial preparation by means of either the natural ovulation cycle or hormone replacement therapy cycles. Embryo transfers were performed under transabdominal ultrasound guidance with the use of IVF catheters (Fuji System) for the embryo transfer. Clinical pregnancy was defined as the presence of a gestational sac, detected by ultrasound.

### 2.4 Spent culture medium collection

When the culture was finished, embryos were removed from the wells and 20 μL of the applied 25 μL of SCM was collected and stored at –20°C. SCM was centrifuged (2500 *g* for 1 minute) to remove potentially contaminated cellular debris and sperm and the supernatant was used for DNA extraction.

### 2.5 Defined blastocysts used in this study

For the present analysis, SCM was used only from blastocyst that reached the expanded blastocyst stage (≥170 µm in inner diameter). Blastocysts with an inner diameter at the <170 µm, hatched and hatching blastocysts, and degeneration blastocysts were excluded.

### 2.6 Morphological evaluation of the blastocysts

Blastocysts were classified as described by Gardner and Schoolcraft,^1^, ^2^ by assigning values from 1 to 6 based on the degree of expansion and hatching status. The quality of the inner cell mass (ICM) and trophectoderm (TE) was also scored. The ICM was graded as (A) many cells tightly packed, (B) several cells loosely grouped, and (C) very few cells, and TE was graded as (A), many cells forming a cohesive epithelium, (B) few cells forming a loose epithelium, and (C) very few large cells.

### 2.7 Morphokinetic evaluation

Images of individual blastocysts were retrospectively analyzed using Embryo Viewer, an external computer workstation (Vitrolife), and the timing of embryonic developmental events during culture from post‐insemination to the expanded blastocyst stage was evaluated as described.^17^ Morphokinetic parameters included pronuclear fading (tPNf), onset of 2‐9 cell divisions (t2, t3, t4, t5, t6, t7, t8, t9+), timing of morula formation (tM), start of blastulation (tSB; first signs of a visible blastocoel), full blastocyst (tB; just before ZP thinning, blastocyst has not reached an inner diameter of 170 µm), and expanded blastocyst (tEB; time of reached an inner diameter of 170 µm). Based on the findings, we determined durations between the following developmental events: period of second cell cycle from 2 to 3 cells (CC2), third cell cycle from 3 to 5 cells (CC3), blastulation from tSB to tB (tB–tSB), duration of blastocyst expansion from tSB to tEB (tEB–tSB), and from tB to tEB (tEB–tB).

### 2.8 DNA extraction and Quantification of cf‐mtDNA copy number

We extracted DNA from the SCM of expanded blastocysts into an equal volume of lysis buffer (final concentration: 20 mM Tris, 0.4 mg/mL proteinase K, 0.9% [v/v], Nonidet P‐40, and 0.9% [v/v] Tween 20), then incubated the solution at 55°C for 30 minutes and 95°C for 5 minutes. Copy numbers of cf‐mtDNA were determined by real‐time PCR using the CFX Connect™ Real‐Time PCR system (Bio‐Rad Laboratories Inc). The primers were designed based on the reference sequence, NC_012920.1, 16 569 bp, derived from the human mitochondrial genome database, and Primer‐BLAST. The primers were 5′‐ccctaaaacccgccacatct ‐3′ and 5′‐ggcctaggttgaggttgacc‐3′, which targeted short (126 bp) mitochondrial sequences (3486‐3612). The PCR cycling program comprised initial denaturation at 95°C for 3 minutes, followed by 40 cycles at 97°C for 6 s and 60°C for 10 s. The PCR efficiency was confirmed using a melting curve and electrophoresis. A standard curve was generated for each run using 10‐fold serial dilutions of representative PCR products that were cloned using Zero Blunt TOPO PCR Cloning Kits (Invitrogen). Copy numbers of standard PCR products were obtained from their concentrations using Avogadro numbers. The PCR products were confirmed by sequencing before use. Based on the copy number of mitochondrial genomes in the sample, cf‐mtDNA copy numbers were calculated per 20 μL of culture medium.

### 2.9 Definition of blastocysts collapse

**FIGURE 1:**
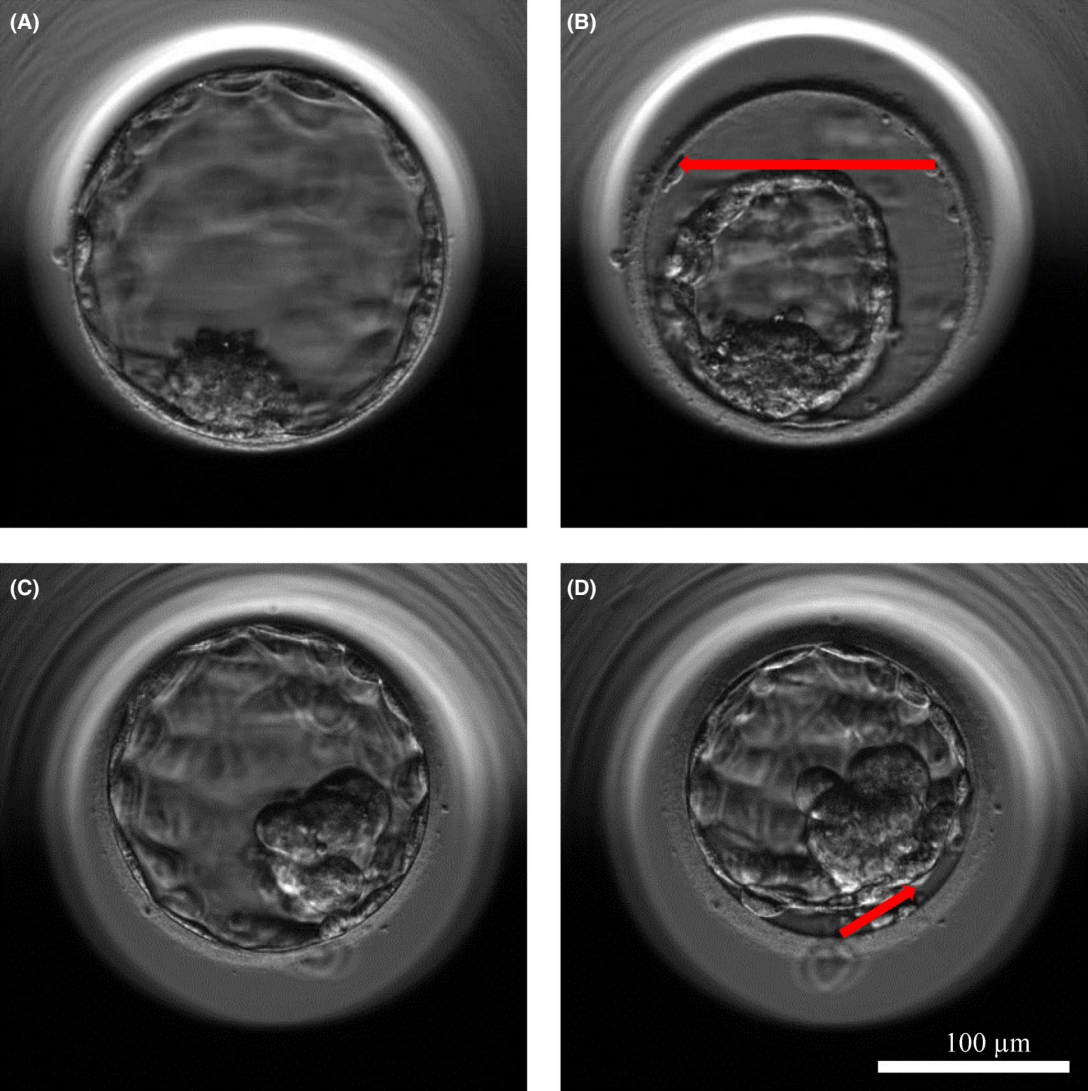
Representative images of human blastocyst collapse (BC) and contraction recorded by time‐lapse monitoring. A, B, embryos with BC. (A) Embryo before BC. (B) Embryo at BC (volume reduction ≥ 50%). C, D, Embryos with contraction but not BC. (C) Embryo before contraction. (D) Embryo at contraction (volume reduction < 50%). Red line, BC and contraction. Bar = 100 μm

Blastocyst collapse (BC) was defined as described by Bodri et al and Sciorio et al.^18^, ^19^ During collapse, volumes of embryos at maximum expansion and of embryos with the lowest volume during collapse were compared using the EmbryoViewer drawing tool. Reductions in embryonic volume of ≥50% and <50% were respectively defined as BC and contraction. Figure 1 shows representative embryos at BC or contraction.

### 2.10 Statistical Analysis

Data were statistically analyzed using JMP statistical software, version 14.0.0 (SAS Institute Inc). Relationships between cf‐mtDNA abundance in the SCM and other explanatory variables (age, BMI [body mass index], AMH [anti‐Müllerian hormone] levels, cumulative ovum pick‐up [OPU], and embryo transfer [ET] cycles) were evaluated using multiple linear regression analyses. The relationship between blastocyst morphological grade and abundance of cf‐mtDNA in SCM was analyzed using Kruskal‐Wallis tests. Correlations between abundance of cf‐mtDNA in SCM and morphokinetic parameters, durations, and numbers of BC were analyzed using Spearman rank‐order correlation coefficients (rho, r). Relationships between cf‐mtDNA abundance in SCM and insemination protocol, as well as the presence or absence of BC were assessed using Mann‐Whitney *U* tests. Values were considered statistically significant at *P* < .05.

## 3 RESULTS

### 3.1 Embryo background and abundance of cf‐mtDNA in the SCM

**FIGURE 2:**
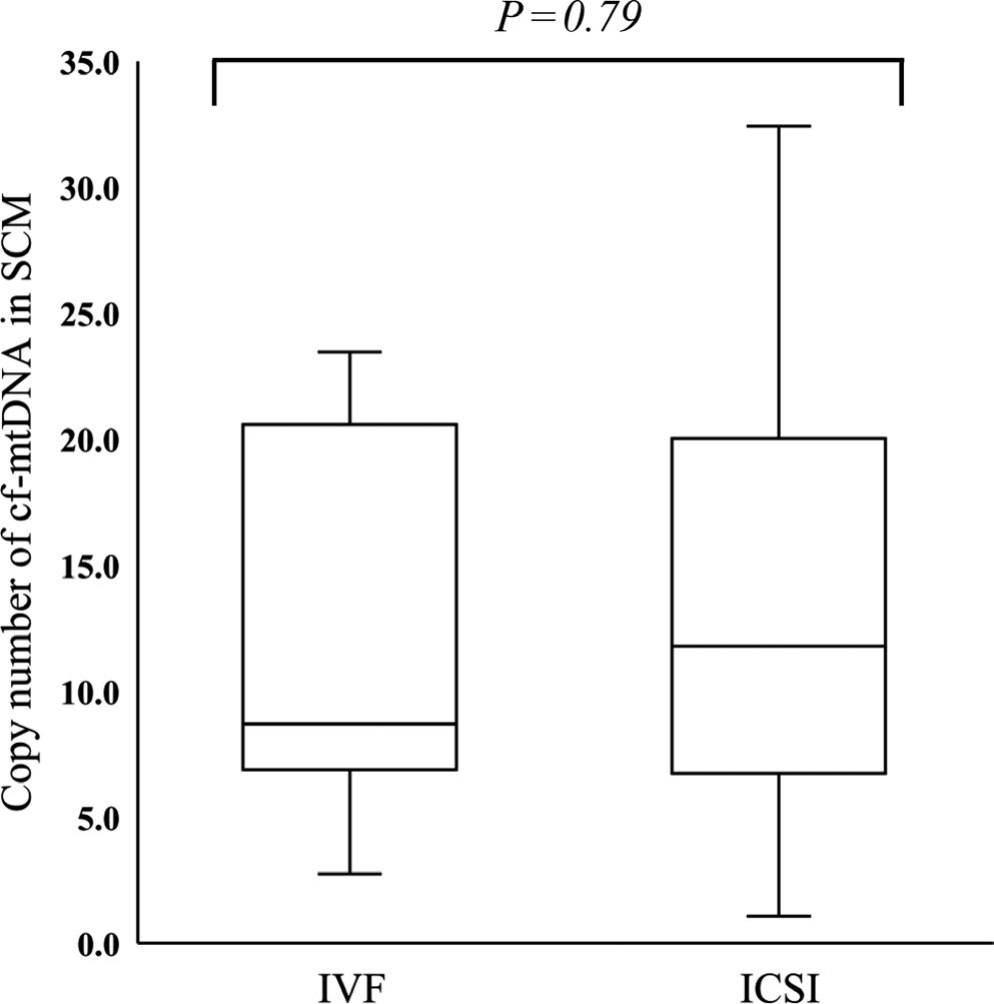
Comparison of cf‐mtDNA abundance in SCM between insemination methods. Cell‐free mitochondrial DNA (cf‐mtDNA) copy numbers in spent culture medium (SCM) of expanded blastocysts were compared between standard in vitro fertilization (IVF: n = 15) and intra‐cytoplasmic sperm injection (ICSI: n = 32). *Y*‐axis, copy number of cf‐mtDNA in 20 μL of SCM *P* = .79

The overall average copy number/20 µL of cf‐mtDNA determined in SCM from 53 blastocysts using real‐time PCR was 13.0. Table 1 shows that mean age (36.0 [31‐44] years), BMI (20.9 [18.9‐25.6] kg/m^2^), AMH level (1.53 [0.03‐5.82] ng/mL), and cumulative numbers of OPU (0.9 [0‐4]) and ET (0.8 [0‐7]) cycles were not significantly associated with cf‐mtDNA abundance in the SCM. Figure 2 shows that the mean (±SD) abundance of cf‐mtDNA in SCM did not differ between standard IVF and ICSI (12.0 ± 6.9 vs. 13.4 ± 7.7, respectively, *P* = .79).

**TABLE 1:** Patient background and association with cf‐mtDNA abundance in SCM

Explanatory variables	Mean	Median	Range	SD	Association with cf‐mtDNA abundance *P* values
Age (years)	36.0	35	31‐44	3.5	.50
BMI (kg/m^2^)	20.9	20.2	18.9‐25.6	1.8	.31
AMH (ng/mL)	1.5	1.5	0.03‐5.8	1.1	.52
Cumulative OPU cycles (times)	0.9	0	0‐4	1.3	.13
Cumulative ET cycles (times)	0.8	0	0‐7	1.8	.94

Note

Associations between abundance of cf‐mtDNA in SCM and age, BMI, AMH level, cumulative OPU, and ET cycles (including frozen‐thawed ET) were analyzed by multiple linear regression models. Data are shown as means, medians, range, and SD.

Abbreviations: AMH, anti‐Müllerian hormone; BMI, body mass index; cf‐mtDNA, cell‐free mitochondrial DNA; ET, embryo transfer; OPU, ovum pick‐up; range, minimum to maximum; SD, standard deviation.

### 3.2 Morphological blastocyst quality and abundance of cf‐mtDNA in the SCM

**FIGURE 3:**
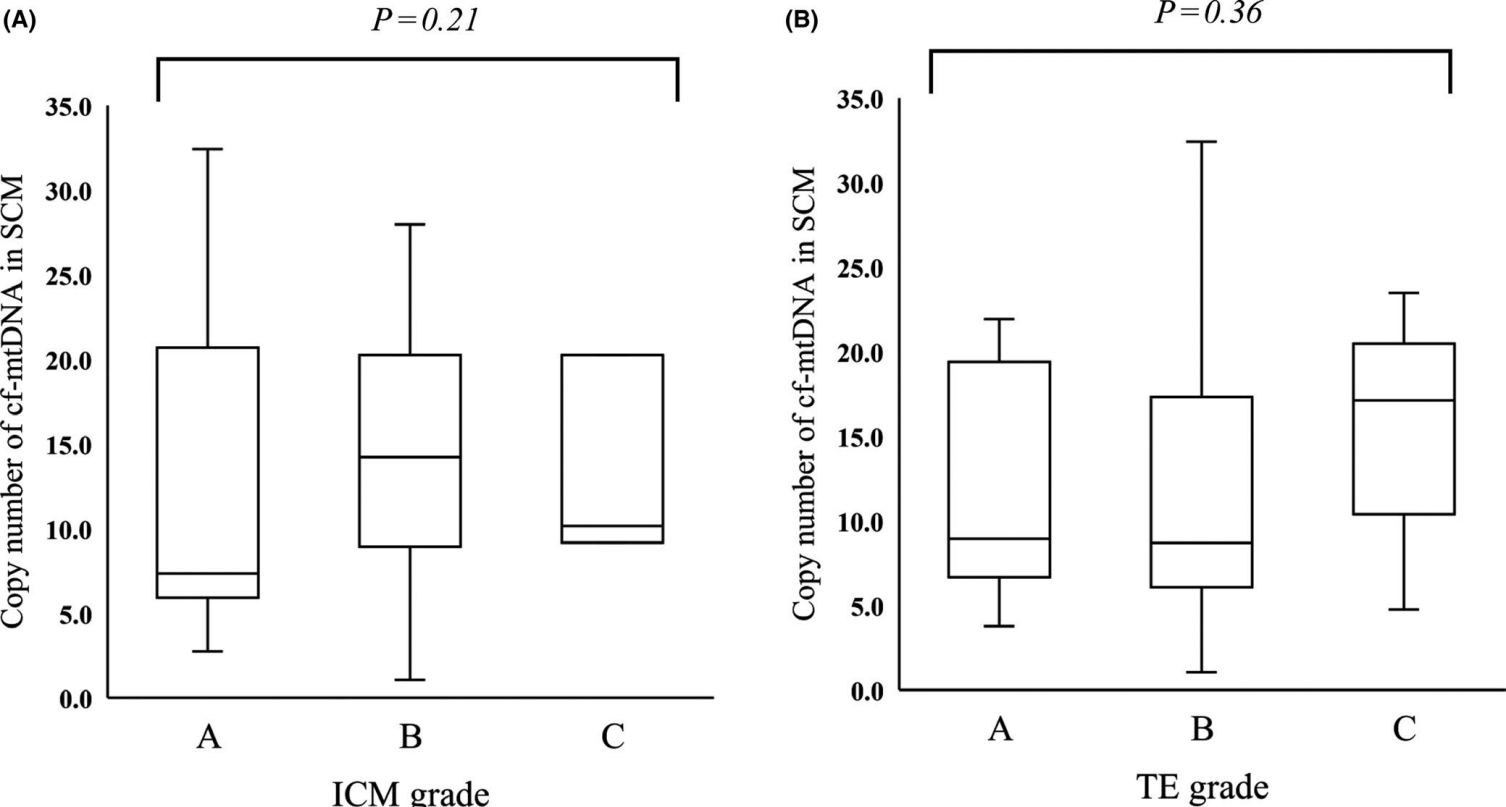
Comparison of cf‐mtDNA abundance in SCM among morphological blastocyst grades. Blastocysts were categorized as grades A, B, or C according to volume and tightly packed cells in ICM and into grades A, B, or C according to numbers and cohesiveness of epithelial cells in TE. SCM of expanded blastocysts was collected, and the cf‐mtDNA copy numbers in SCM were compared among ICM grades (A, n = 27; B, n = 23; C, n = 3; *P* = .21) or TE grades (A, n = 13; B, n = 28; C, n = 12; *P* = .36). *Y*‐axis, copy numbers of cf‐mtDNA in 20 μL of SCM. cf‐mtDNA, cell‐free mitochondrial DNA; ICM, inner cell mass; SCM, spent culture medium; TE, trophectoderm

Mean (±SD) cf‐mtDNA abundance in the SCM was not affected by embryonic grade (ICM grades A, B, and C, respectively: 11.9 ± 8.0, 14.2 ± 6.8, and 13.2 ± 5.0, *P* = .31 [Figure 3A]; TE grades A, B, and C, respectively: 12.1 ± 6.4, 12.1 ± 8.2, and 15.9 ± 5.8, *P* = .20 [Figure 3B]).

### 3.3 Morphokinetic data and abundance of cf‐mtDNA in the SCM

The cf‐mtDNA abundance in the SCM did not significantly correlate with any morphokinetic parameters during all incubation periods (Table 2). However, duration of tEB–tSB (r = .46, *P* < .01) and tEB–tB (r = .47, *P* < .01) significantly and positively correlated with the abundance of cf‐mtDNA in the SCM (Table 3).

**TABLE 2:** Correlation between morphokinetic parameters and cf‐mtDNA abundance in SCM

Morphokinetic parameters	Mean (range) h	Correlation with cf‐mtDNA abundance (*r*)	*P* values
*tPNf*	24.7 (19.5‐33.4)	−.11	.45
*t2*	27.2 (21.6‐35.9)	−.07	.63
*t3*	39.8 (26.8‐53.4)	−.08	.60
*t4*	41.5 (33.3‐55.2)	−.16	.28
*t5*	53.8 (34.1‐74.1)	−.10	.50
*t6*	56.6 (38.6‐76.1)	−.12	.42
*t7*	60.0 (38.6‐85.4)	−.06	.67
*t8*	64.6 (47.8‐86.4)	−.08	.58
*t9+*	76.0 (49.8‐95.6)	−.24	.11
*tM*	98.0 (73.4‐128.9)	−.08	.58
*tSB*	106.9 (88.6‐137.1)	−.26	.08
*tB*	113.6 (94.1‐139.7)	−.28	.05
*tEB*	128.3 (107.8‐160.2)	−.05	.75

Note

Data are shown as means, range, correlation coefficient and *P* value. Abbreviations: h, hours; *r*, Spearman rank‐order correlation coefficient; tB, full blastocyst; tEB, expanded blastocyst; tM, morula formation; tPNf, pronuclear fading; tSB start of blastulation; t2, t3, t4, t5, t6, t7, t8, t9+, onset of 2 −9 cell divisions.

**TABLE 3:** Correlation between morphokinetic durations and cf‐mtDNA abundance in SCM

Morphokinetic durations	Mean (range) h	Correlation with cf‐mtDNA abundance (*r*)	*P* values
*t3‐t2 (CC2)*	12.6 (3.3‐17.5)	−.14	.34
*t5‐t3 (CC3)*	14.0 (0.0‐25.6)	−.12	.45
*tB‐tSB*	6.7 (2.0‐17.5)	−.06	.68
*tEB‐tSB*	21.4 (8.3‐38.0)	−.46	<.01
*tEB‐tB*	14.7 (5.0‐27.7)	−.47	<.01

Note

Data are shown as means, range, correlation coefficient, and *P* value.

Abbreviations: CC2, second cell cycle from 2 to 3 cells; CC3, third cell cycle from 3 to 5 cells; h, hours; *r*, Spearman rank‐order correlation coefficient; tB–tSB, duration of tSB to tB; tEB‐tB, duration of tB to tEB; tEB–tSB, duration of tSB to tEB.

### 3.4 Blastocyst collapse and abundance of cf‐mtDNA in the SCM

**FIGURE 4:**
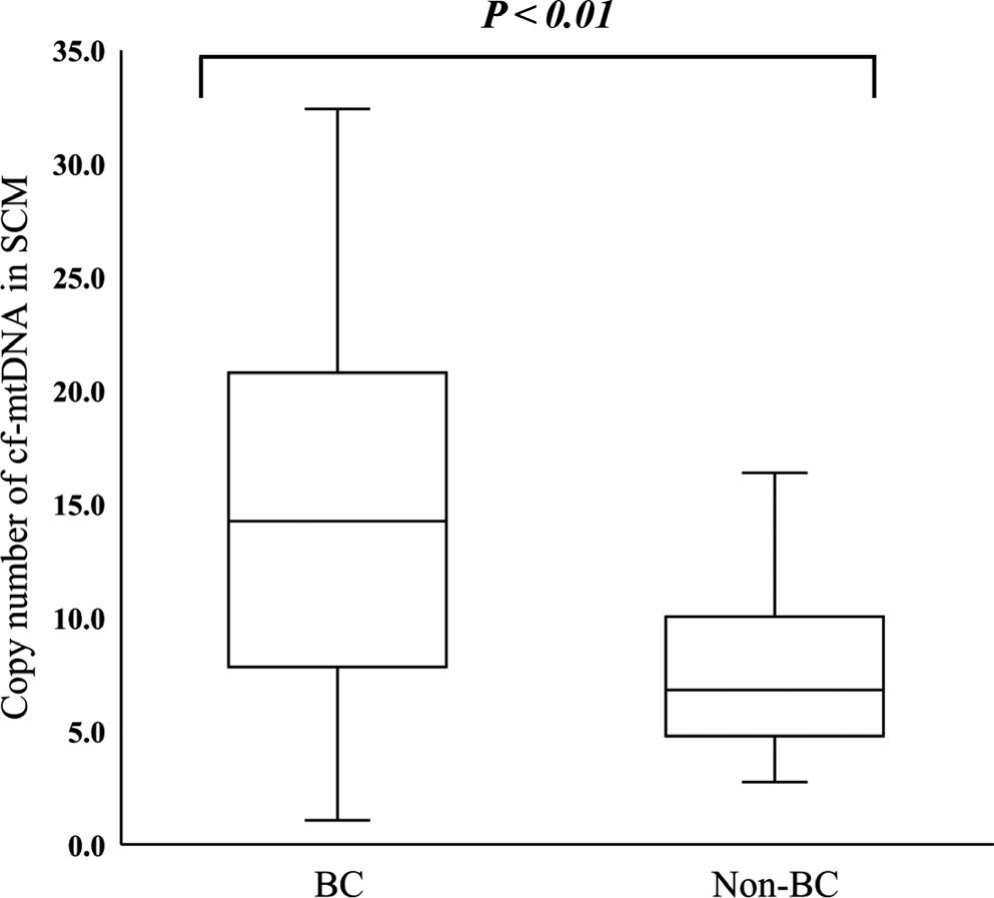
Comparison of cf‐mtDNA abundance in SCM between blastocysts with and without BC. Cf‐mtDNA copy numbers in SCM were compared between blastocysts with (BC; n = 39) and without (Non‐BC; n = 14) BC. *Y*‐axis, copy number of cf‐mtDNA in 20 μL of SCM *P* < .01. BC, blastocyst collapse; cf‐mtDNA, cell‐free mitochondrial DNA; SCM, spent culture medium

**FIGURE 5:**
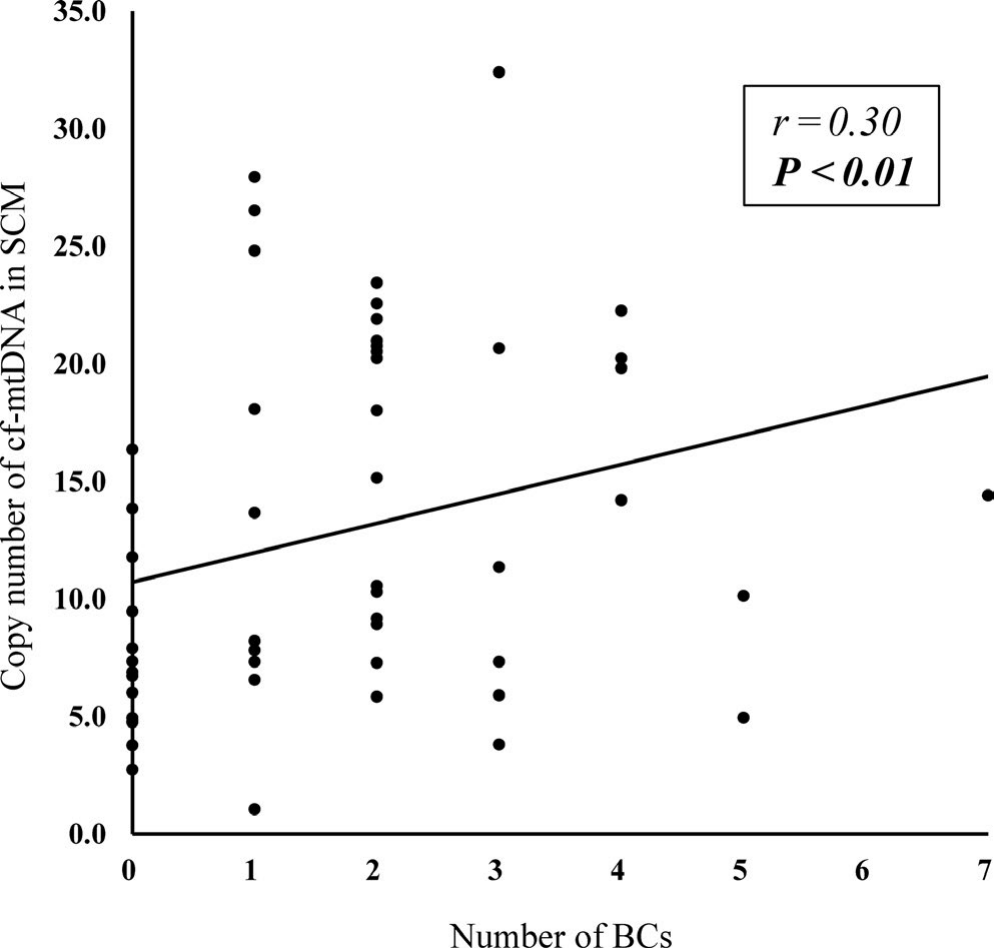
Relationship between abundance of cf‐mtDNA in SCM and number of BC. *Y*‐axis, copy number of cf‐mtDNA in 20 μL of SCM. *X*‐axis, number of BC episode in corresponding expanded blastocyst. Spearman coefficient (*r*), .30. *P* < .01. BC, blastocyst collapse; cf‐mtDNA, cell‐free mitochondrial DNA; SCM, spent culture medium

Figure 4 shows that the mean (±SD) cf‐mtDNA abundance in SCM was significantly greater in blastocysts with, than without BC (14.9 ± 7.7 vs. 7.67 ± 3.9, *P* < .01), and that it significantly correlated with the number of BC (r = .31, *P* < .01) (Figure 5).

### 3.5 Blastocyst collapse and duration of expansion

**FIGURE 6:**
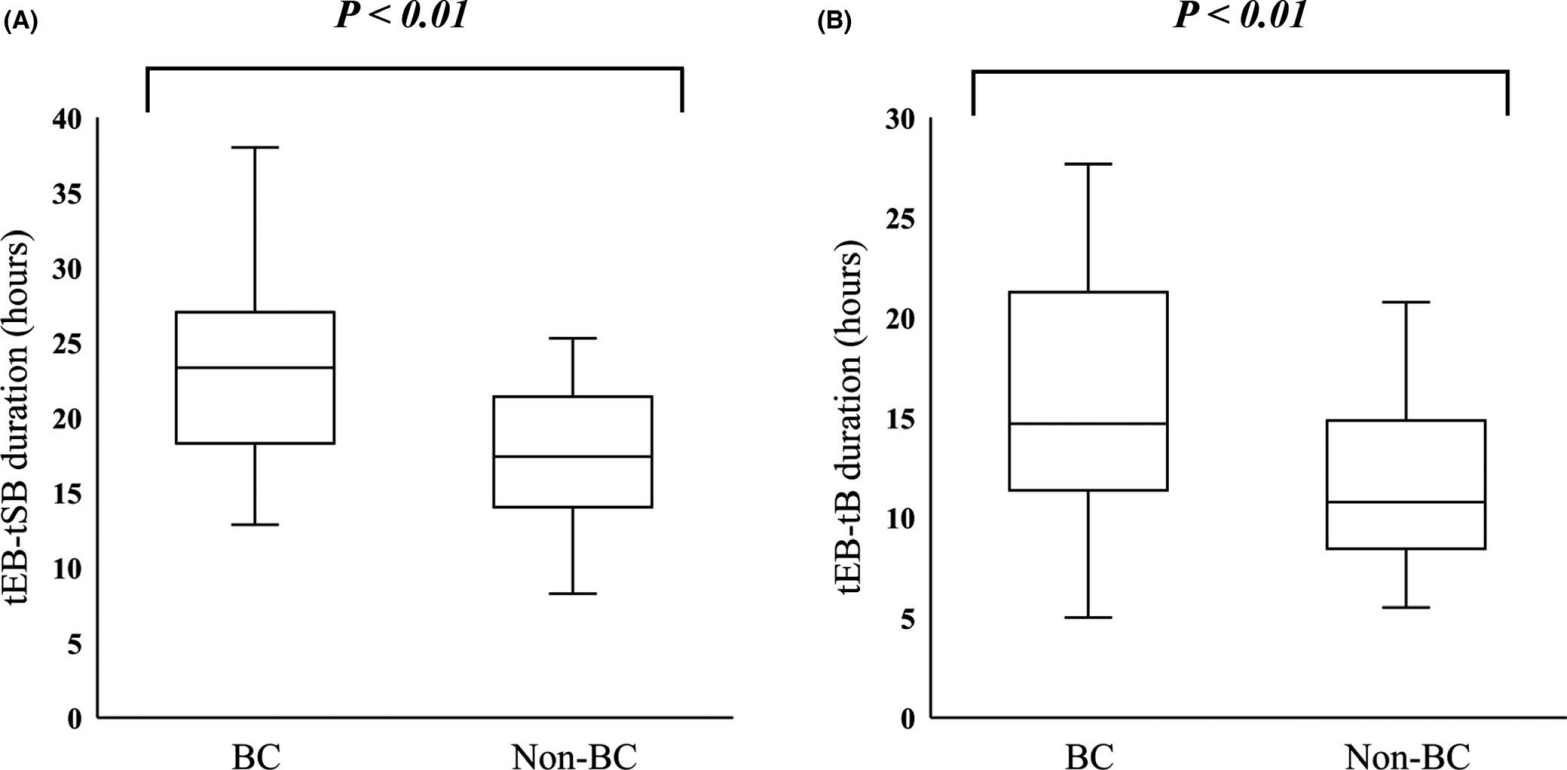
Comparison of tEB–tSB and tEB–tB durations between blastocysts with and without BC. Boxplots show blastocysts with (BC; n = 39) and without (Non‐BC; n = 14) BC. *Y*‐axis, durations between tEB–tSB (A) and tEB–tB (B) (hours). *P* < .01. BC, blastocyst collapse; tEB–tB, duration between blastocyst stage (tB) and expanded blastocyst (tEB); tEB–tSB, duration between start of blastulation (tSB) and expanded blastocyst (tEB)

Figure 6A,B shows relationships between BC and the duration of the blastocyst stage in embryos with and without BC. The mean (±SD) duration of tEB–tSB (22.9 ± 6.0 vs. 17.2 ± 4.5, *P* < .01) and tEB–tB (15.9 ± 5.9 vs. 11.3 ± 4.1, *P* < .01) was significantly longer in embryos with, than those without BC.

### 3.6 Embryonic quality, culture duration, and cf‐mtDNA in the SCM

**FIGURE 7:**
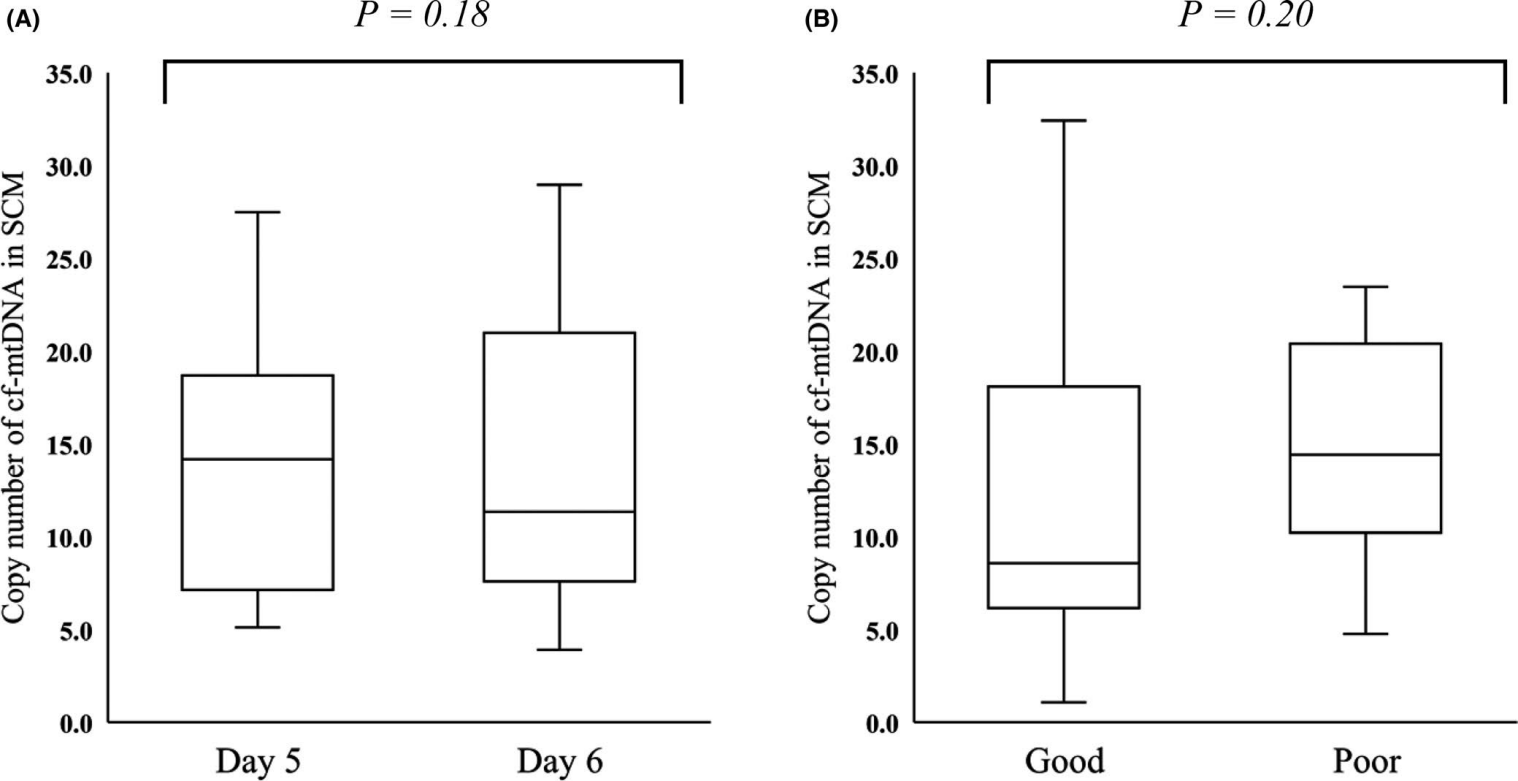
Comparison of cf‐mtDNA abundance in SCM at day 5 and day 6 and between good and poor blastocysts. A, Cell‐free mitochondrial DNA (cf‐mtDNA) copy numbers in spent culture medium (SCM) were compared between SCM at culture day 5 (n = 18) and culture day 6 (n = 35). *Y*‐axis, copy number of cf‐mtDNA in 20 μL of SCM *P* = .18. B, Cell‐free mtDNA copy numbers in SCM were compared between morphologically good (AA, AB, BA and BB, n = 40) and poor (BC and CC, n = 13) blastocysts. *Y*‐axis, copy number of cf‐mtDNA in 20 μL of SCM *P* = .20. cf‐mtDNA, cell‐free mitochondrial DNA; SCM, spent culture medium

**FIGURE 8:**
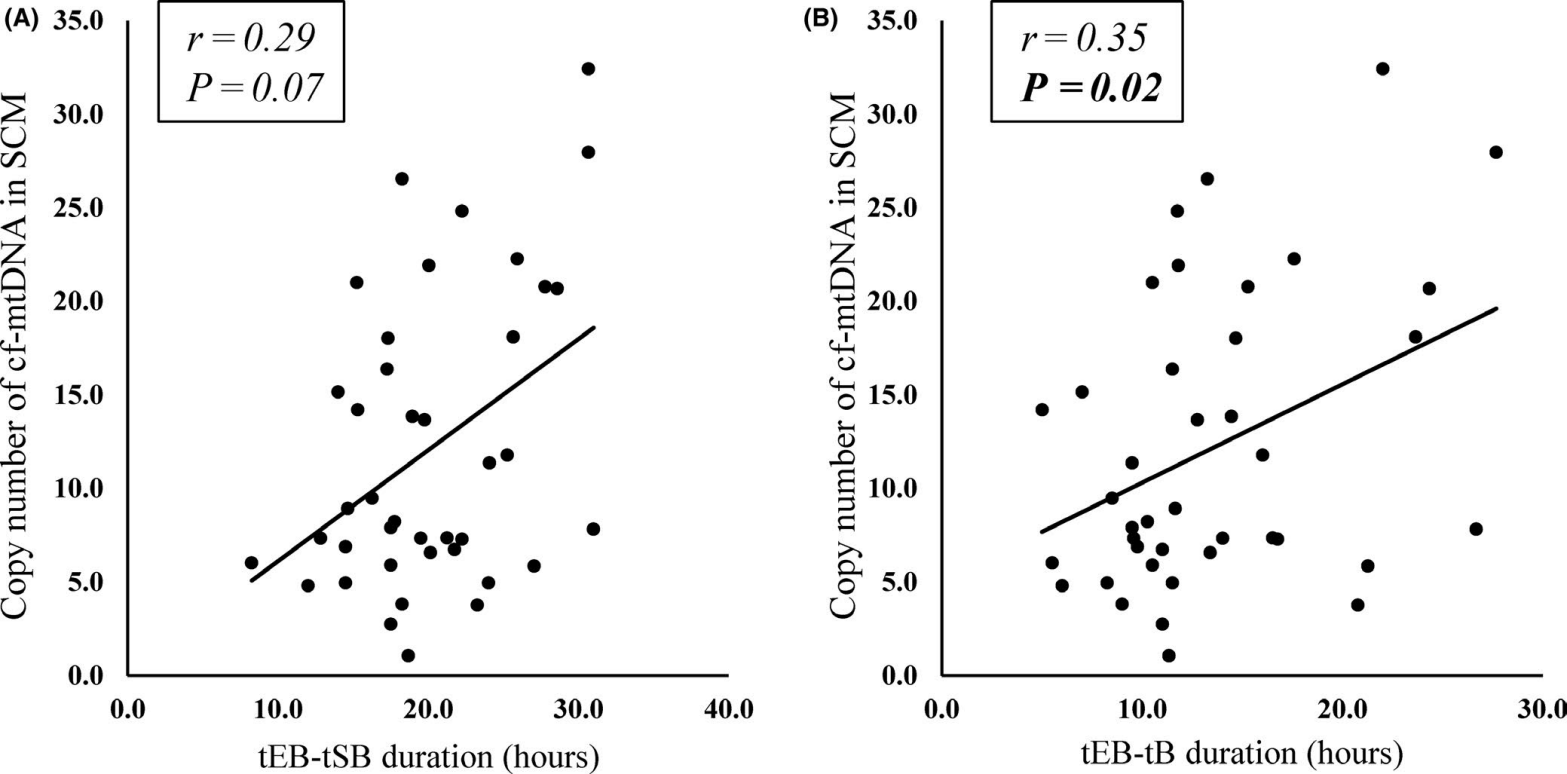
Correlation between cf‐mtDNA abundance in SCM and tEB–tSB and tEB–tB durations using data from morphologically good blastocysts. A, *Y*‐axis, copy number of cf‐mtDNA in 20 μL of SCM. *X*‐axis, tEB‐tSB durations (hours). Spearman coefficient (*r*), .29. *P* = .07. B, *Y*‐axis, copy number of cf‐mtDNA in 20 μL of SCM. *X*‐axis, tEB‐tB durations (hours). Spearman coefficient (*r*), .35. *P* = .02. cf‐mtDNA, cell‐free mitochondrial DNA; SCM, spent culture medium; tEB–tB, duration between blastocyst stage (tB), and expanded blastocyst (tEB); tEB–tSB, duration between start of blastulation (tSB) and expanded blastocyst (tEB)

**FIGURE 9:**
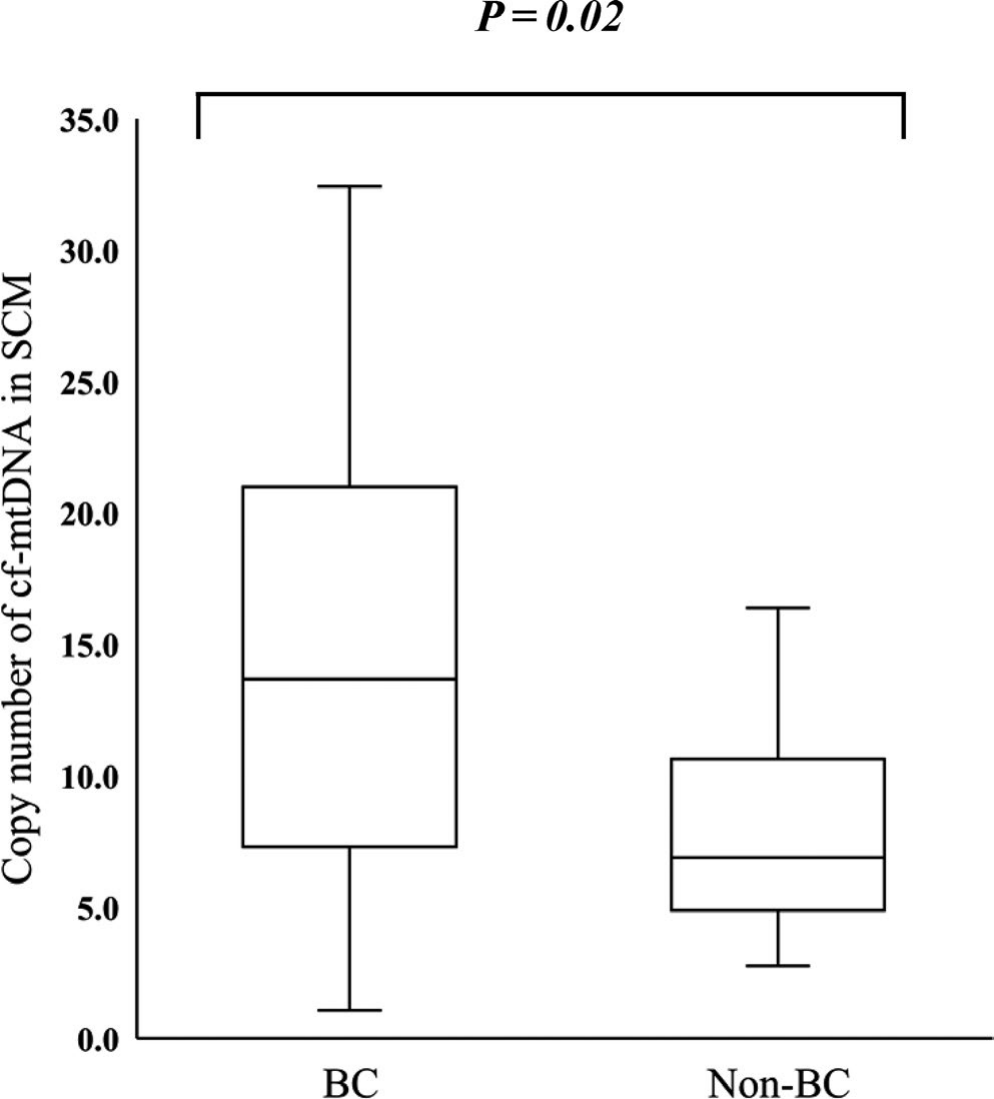
Comparison of cf‐mtDNA abundance in SCM between blastocysts with and without BC using the data from good blastocysts. Cf‐mtDNA copy numbers of SCM were compared between blastocysts with BC (BC; n = 27) and without BC (Non‐BC; n = 13). *Y*‐axis, copy number of cf‐mtDNA in 20 μL of SCM *P* = .02. BC, blastocyst collapse; cf‐mtDNA, cell‐free mitochondrial DNA; SCM, spent culture medium

Here, we addressed the question of whether the greater abundance of cf‐mtDNA in the SCM was caused by longer culture duration or poor morphological quality of the blastocyst. A significant difference in the copy number if cf‐mtDNA in the SCM was not observed between day 5 blastocyst and day 6 blastocyst (11.2 ± 7.2 vs. 13.9 ± 7.7, *P* = .18; Figure 7A) or between good blastocysts (AA, AB, BA, and BB grade) and poor blastocysts (BC and CC grade) (12.3 ± 7.4 vs. 15.5 ± 6.3, *P* = .20; Figure 7B). Furthermore, a significant positive correlation was observed between the cf‐mtDNA content in SCM and tEB‐tB duration in good blastocysts (r = .35, *P* = .025; Figure 8B). While the correlation was not significant, there was also a positive trend between cf‐mtDNA content and tEB‐tSB duration (r = .28, *P* = .07; Figure 8A). Moreover, good blastocyst with BC had a greater abundance of cf‐mtDNA in the SCM than those without BC (14.9 ± 7.7 vs. 7.67 ± 3.9, *P* = .02; Figure 9).

### 3.7 Clinical pregnancy and abundance of cf‐mtDNA in the SCM

Twenty‐eight blastocysts with abundant cf‐mtDNA in the SCM were frozen‐thawed and transferred, and eight of these gave rise to clinical pregnancy. SCM of embryos that successfully implanted contained 11.9 ± 7.3 copies of cf‐mtDNA (n = 8), whereas SCM of embryos that failed to implant contained 15.5 ± 8.1 copies (n = 20); this difference was not significant (*P* = .33). We added this dataset in the result section. In addition, we classified the SCM into two groups according to the mean of cf‐mtDNA abundance (14.5); Large and Small cf‐mtDNA groups. Clinical pregnancy rate was 23.1% (3/13) for large group and 33.3% (5/15) for small group (*P* > .05).

## 4 DISCUSSION

The present study demonstrated significant relationships between abundance of cf‐mtDNA in the SCM and morphokinetic parameters of the embryos including duration of blastocyst stages and presence of blastocyst collapse which were obtained using time‐lapse incubator.

The physiological status of patients considerably affects the quality of embryos. Therefore, we investigated whether patient status affects the abundance of cf‐mtDNA in SCM. We confirmed that the abundance of cf‐mtDNA in SCM was not associated with the age, or the BMI, AMH level, or cumulative OPU and ET cycles of patient. These results indicate that patient‐associated factors did not strongly affect cf‐mtDNA abundance in the SCM in the present study. Morphokinetic parameters are associated with chromosome abnormalities and clinical outcomes after embryo transfer. Campbell et al associated delayed tSB and tB with high rates of embryonic aneuploidy and established cutoff timing for normal embryonic development as <96.6 and <118.1 hours, respectively.^20^ Consistent with this, Desai et al reported that early expanded blastocyst stage, ≤116.0 hours (tEB), and shorter duration of blastocyst expansion, ≤13.0 hours (tEB–tSB), are markers of high likelihood of euploid embryos.^21^ The present study found that cf‐mtDNA abundance in the SCM significantly and positively correlated with the duration of tEB–tSB and of tEB–tB. In addition, when the data were filtered by embryonic grade, the difference was also observed in good blastocysts. These results indicate that higher abundance of cf‐mtDNA in the SCM is a marker of poor blastocyst quality.

The major unresolved issues regarding cf‐mtDNA in the SCM are its origin and its secretion mechanism from embryos into the SCM. That is, cf‐mtDNA might be generated from dead or fragmented blastomeres or arise due to specific events in blastocysts or actively secreted blastocysts.

Furthermore, the present study found no significant differences in cf‐mtDNA abundance in the SCM with respect to morphological blastocyst quality (ICM and TE grades).

However, whether cf‐mtDNA abundance in the SCM reflects mitochondrial numbers in corresponding blastocysts remains to be clarified.

More mitochondria in oocytes is a marker of good quality, as oocytes with fewer mitochondria have poor fertilization and developmental outcomes.^22^, ^23^ However, this is not true of blastocysts, as mtDNA abundance in TE cells and the quality of blastocysts are negatively associated; that is, mtDNA is more abundant in TE cells in aneuploid blastocysts.^24^, ^25^ After fertilization, the number of mitochondria in embryos decreases and those in embryos at the early developmental stage reflect the number in oocytes, because active mitochondria are not generated until the blastocyst stage.^26^, ^27^, ^28^ We did not assess relationships between mitochondrial DNA copy numbers in blastocysts and cf‐mtDNA abundance in the SCM, but higher mtDNA content in blastocysts might be the background for the increased abundance of cf‐mtDNA in the corresponding SCM. The notion that cf‐mtDNA abundance reflects poor embryo quality contradicts that reported for cf‐mtDNA in the SCM of early cleaved embryos. Stigliani et al^29^ associated abundance of cf‐mtDNA in the SCM of early cleaved embryos (Day 3) with good blastocyst formation and implantation rates. Stigliani et al^30^ also summarized data derived from multi‐center studies and found higher cf‐mtDNA abundance in the SCM of embryos derived from younger, than older patients. Therefore, they concluded that cf‐mtDNA abundance in the SCM of early stage embryos is a positive predictive marker of embryo development and clinical outcome. Hammond et al also found more cf‐mtDNA in the SCM in day 5 and 6 embryos than in the medium of day 3 embryos, showing that embryos stage‐specifically secrete cf‐mtDNA.^31^ We targeted cf‐mtDNA abundance in the SCM at the expanded blastocyst stage on days 5 or 6. Thus, the different developmental stage could explain the contradictory conclusions. The present study identified a positive association between cf‐mtDNA abundance in SCM and the presence and frequency of BC, and confirmed longer tEB–tSB and tEB–tB duration in blastocysts with, than without BC. Physiological stress, excessive energy expenditure, and dysfunctional cellular gap junctions reportedly cause blastocysts to collapse.^32^ Furthermore, BC is a hallmark of poor embryo quality, which results in low CPR and LBR in human blastocyst transfer.^17^, ^18^, ^33^ These and the present results suggest that BC is one cause of delayed blastocyst expansion, and that BC increases the abundance of cf‐mtDNA in the SCM.

Blastocoel fluid (BF) has recently been identified as a source of embryonic DNA for noninvasive PGT. Gianaroli et al^34^ considered that BF could represent an alternative source of embryonic DNA material for chromosomal tests because it can accurately predict embryo ploidy and chromosome content. Xu et al^35^ found a high chromosome concordance rate between prediction based on SCM and on corresponding blastocysts. Here, the mitochondrial genome was easily detected in the SCM, but the copy number of nucleic DNA was lower than that in the mitochondrial genome (data not shown). These data are consistent with previous findings showing up to several hundred‐fold more mitochondrial genomic DNA than nuclear DNA in the SCM.^14^

The main limitation of the present study is the sample size, which was too small to demonstrate a statistically significant relationship between cf‐mtDNA abundance in SCM and clinical outcomes. However, we found a trend toward greater abundance of cf‐mtDNA in the SCM of embryos that failed to implant. In addition, the data obtained on culture duration and embryonic stage improve the understanding of the significance of cf‐mtDNA and should be examined further in future studies.

In conclusion, this study revealed more abundant cf‐mtDNA in the SCM of embryos with delayed blastocyst expansion and BC. This suggests that cf‐mtDNA abundance in the SCM could serve as an additional or alternative marker of blastocyst evaluation.

## DISCLOSURES

*Conflicts of interest*: The authors declare no conflict of interest. *Human rights statements, clinical trials, and informed consent*: Sample collection and all experiments were conducted at Kanagawa Ladies Clinic by M. Kobayashi and J. Kobayashi, and analysis of data, and manuscript writing was conducted at Tokyo University of Agriculture by M. Kobayashi, K Shirasuna, and H. Iwata. All procedures complied with the ethical standards of the Institutional Ethics Committee at Kanagawa Ladies Clinic, Kanagawa, Japan, and with the Declaration of Helsinki (2013). All patients in this study provided informed consent to participate (approved No. 003). This was a retrospective observational study and we used only image recorded by time‐lapse incubator and spent culture medium after embryo culture and did not include any invasive clinical trials. This study did not include experimental animals. *Animal studies*: This study does not include animal experiments. *Approval by Ethical Committee*: All procedures complied with the ethical standards of the Institutional Ethics Committee at Kanagawa Ladies Clinic, Kanagawa, Japan, and with the Declaration of Helsinki (2013). *Clinical Trials registration*: This study does not include clinical trials.

## References

[1] Gardner DK , Lane M , Stevens J , Schlenker T , Schoolcraft WB . Blastocyst score affects implantation and pregnancy outcome: towards a single blastocyst transfer. Fertil Steril. 2000;73:1155‐1158. 10856474 10.1016/s0015-0282(00)00518-5

[2] Schoolcraft WB , Gardner DK , Lane M , Schlenker T , Hamilton F , Meldrum DR . Blastocyst culture and transfer: analysis of results and parameters affecting outcome in two in vitro fertilization programs. Fertil Steril. 1999;72:604‐609. 10521095 10.1016/s0015-0282(99)00311-8

[3] Storr A , Venetis CA , Cooke S , Kilani S , Ledger W . Inter‐observer and intra‐observer agreement between embryologists during selection of a single Day 5 embryo for transfer: a multicenter study. Hum Reprod. 2017;32:307‐314. 28031323 10.1093/humrep/dew330

[4] Yang ST , Shi JX , Gong F , et al. Cleavage pattern predicts developmental potential of day 3 human embryos produced by IVF. Reprod Biomed Online. 2015;30:625‐634. 25892500 10.1016/j.rbmo.2015.02.008

[5] Fan YL , Han SB , Wu LH , Wang YP , Huang GN . Abnormally cleaving embryos are able to produce live births: a time‐lapse study. J Assist Reprod Genet. 2016;33:379‐385. 26749387 10.1007/s10815-015-0632-x PMC4785157

[6] Kirkegaard K , Sundvall L , Erlandsen M , Hindkjær JJ , Knudsen UB , Ingerslev HJ . Timing of human preimplantation embryonic development is confounded by embryo origin. Hum Reprod. 2016;31:324‐331. 26637491 10.1093/humrep/dev296 PMC4716807

[7] Campbell A , Fishel S , Laegdsmand M . Aneuploidy is a key causal factor of delays in blastulation: author response to “A cautionary note against aneuploidy risk assessment using time‐lapse imaging”. Reprod Biomed Online. 2014;28:279‐283. 24444816 10.1016/j.rbmo.2013.11.016

[8] Lee HL , McCulloh DH , Hodes‐Wertz B , Adler A , McCaffrey C , Grifo JA . In vitro fertilization with preimplantation genetic screening improves implantation and live birth in women age 40 through 43. J Assist Reprod Genet. 2015;32:435‐444. 25578536 10.1007/s10815-014-0417-7 PMC4363234

[9] Forman EJ , Hong KH , Ferry KM , et al. In vitro fertilization with single euploid blastocyst transfer: A randomized controlled trial. Fertil Steril. 2013;100:100‐107.e1. 23548942 10.1016/j.fertnstert.2013.02.056

[10] Maxwell SM , Colls P , Hodes‐Wertz B , et al. Why do euploid embryos miscarry? A case‐control study comparing the rate of aneuploidy within presumed euploid embryos that resulted in miscarriage or live birth using next‐generation sequencing. Fertil Steril. 2016;106:1414‐1419.e5. 27692437 10.1016/j.fertnstert.2016.08.017

[11] Vera‐Rodriguez M , Diez‐Juan A , Jimenez‐Almazan J , et al. Origin and composition of cell‐free DNA in spent medium from human embryo culture during preimplantation development. Hum Reprod. 2018;33:745‐756. 29471395 10.1093/humrep/dey028

[12] Kansaku K , Munakata Y , Shirasuna K , Kuwayama T , Iwata H . Mitochondrial cell‐free DNA secreted from porcine granulosa cells. Zygote. 2019;27:272‐278. 31411132 10.1017/S096719941900025X

[13] Ichikawa K , Shibahara H , Shirasuna K , Kuwayama T , Iwata H . Cell‐free DNA content in follicular fluid: a marker for the developmental ability of porcine oocytes. Reprod Med Biol. 2020;19:95‐103. 31956291 10.1002/rmb2.12309 PMC6955585

[14] Kansaku K , Munakata Y , Itami N , Shirasuna K , Kuwayama T , Iwata H . Mitochondrial dysfunction in cumulus‐oocyte complexes increases cell‐free mitochondrial DNA. J Reprod Dev. 2018;64:261‐266. 29618676 10.1262/jrd.2018-012 PMC6021605

[15] Hara T , Kin A , Aoki S , et al. Resveratrol enhances the clearance of mitochondrial damage by vitrification and improves the development of vitrifiedwarmed bovine embryos. PLoS One. 2018;13:1‐17. 10.1371/journal.pone.0204571 PMC6193637 30335749

[16] Stigliani S , Persico L , Lagazio C , Anserini P , Venturini PL , Scaruffi P . Mitochondrial DNA in Day 3 embryo culture medium is a novel, non‐invasive biomarker of blastocyst potential and implantation outcome. Mol Hum Reprod. 2014;20:1238‐1246. 25232043 10.1093/molehr/gau086

[17] Mumusoglu S , Yarali I , Bozdag G , et al. Time‐lapse morphokinetic assessment has low to moderate ability to predict euploidy when patient– and ovarian stimulation–related factors are taken into account with the use of clustered data analysis. Fertil Steril. 2017;107:413‐421.e4. 27939508 10.1016/j.fertnstert.2016.11.005

[18] Bodri D , Sugimoto T , Yao Serna J , Kawachiya S , Kato R , Matsumoto T . Blastocyst collapse is not an independent predictor of reduced live birth: a time‐lapse study. Fertil Steril. 2016;105:1476‐1483.e3. 26940789 10.1016/j.fertnstert.2016.02.014

[19] Sciorio R , Thong KJ , Pickering SJ . Spontaneous blastocyst collapse as an embryo marker of low pregnancy outcome: a time‐lapse study. J Bras Reprod Assist. 2020;24:34‐40. 10.5935/1518-0557.20190044 PMC6993169 31397550

[20] Campbell A , Fishel S , Bowman N , et al. Modelling a risk classification of aneuploidy in human embryos using non‐invasive morphokinetics. Reprod Biomed Online. 2013;26:477‐485. 23518033 10.1016/j.rbmo.2013.02.006

[21] Desai N , Goldberg JM , Austin C , Falcone T . Are cleavage anomalies, multinucleation, or specific cell cycle kinetics observed with time‐lapse imaging predictive of embryo developmental capacity or ploidy? Fertil Steril. 2018;109:665‐674. 29452698 10.1016/j.fertnstert.2017.12.025

[22] Reynier P , May‐Panloup P , Chrétien MF , et al. Mitochondrial DNA content affects the fertilizability of human oocytes. Mol Hum Reprod. 2001;7:425‐429. 11331664 10.1093/molehr/7.5.425

[23] Zeng HT , Ren Z , Yeung WSB , et al. Low mitochondrial DNA and ATP contents contribute to the absence of birefringent spindle imaged with PolScope in in vitro matured human oocytes. Hum Reprod. 2007;22:1681‐1686. 17449512 10.1093/humrep/dem070

[24] Fragouli E , Spath K , Alfarawati S , et al. Altered levels of mitochondrial DNA are associated with female age, aneuploidy, and provide an independent measure of embryonic implantation potential. PLoS Genet. 2015;11:1‐18. 10.1371/journal.pgen.1005241 PMC4454688 26039092

[25] Diez‐Juan A , Rubio C , Marin C , et al. Mitochondrial DNA content as a viability score in human euploid embryos: less is better. Fertil Steril. 2015;104:534‐541.e1. 26051102 10.1016/j.fertnstert.2015.05.022

[26] Hashimoto S , Morimoto N , Yamanaka M , et al. Quantitative and qualitative changes of mitochondria in human preimplantation embryos. J Assist Reprod Genet. 2017;34:573‐580. 28190213 10.1007/s10815-017-0886-6 PMC5427646

[27] Lee WTY , John JS . The control of mitochondrial DNA replication during development and tumorigenesis. Ann N Y Acad Sci. 2015;1350:95‐106. 26335356 10.1111/nyas.12873

[28] Pikó L , Taylor KD . Amounts of mitochondrial DNA and abundance of some mitochondrial gene transcripts in early mouse embryos. Dev Biol. 1987;123:364‐374. 2443405 10.1016/0012-1606(87)90395-2

[29] Stigliani S , Anserini P , Venturini PL , Scaruffi P . Mitochondrial DNA content in embryo culture medium is significantly associated with human embryo fragmentation. Hum Reprod. 2013;28:2652‐2660. 23887072 10.1093/humrep/det314

[30] Stigliani S , Orlando G , Massarotti C , et al. Non‐invasive mitochondrial DNA quantification on day 3 predicts blastocyst development: a prospective, blinded, multi‐centric study. Mol Hum Reprod. 2019;25:527‐537. 31174207 10.1093/molehr/gaz032

[31] Hammond ER , McGillivray BC , Wicker SM , et al. Characterizing nuclear and mitochondrial DNA in spent embryo culture media: genetic contamination identified. Fertil Steril. 2017;107:220‐228.e5. 27865449 10.1016/j.fertnstert.2016.10.015

[32] Togashi K , Kumagai J , Sato E , et al. Dysfunction in gap junction intercellular communication induces aberrant behavior of the inner cell mass and frequent collapses of expanded blastocysts in mouse embryos. J Assist Reprod Genet. 2015;32:969‐976. 25917498 10.1007/s10815-015-0479-1 PMC4491087

[33] Marcos J , Pérez‐Albalá S , Mifsud A , Molla M , Landeras J , Meseguer M . Collapse of blastocysts is strongly related to lower implantation success: a time‐lapse study. Hum Reprod. 2015;30:2501‐2508. 26355116 10.1093/humrep/dev216

[34] Gianaroli L , Magli MC , Pomante A , et al. Blastocentesis: a source of DNA for preimplantation genetic testing. Results from a pilot study. Fertil Steril. 2014;102:1692‐1699.e6. 25256935 10.1016/j.fertnstert.2014.08.021

[35] Xu J , Fang R , Chen L , et al. Noninvasive chromosome screening of human embryos by genome sequencing of embryo culture medium for in vitro fertilization. Proc Natl Acad Sci USA. 2016;113:11907‐11912. 27688762 10.1073/pnas.1613294113 PMC5081593

